# Barriers and facilitators to weight management in overweight and obese women living in Australia with PCOS: a qualitative study

**DOI:** 10.1186/s12902-019-0434-8

**Published:** 2019-10-23

**Authors:** Siew Lim, Caroline A. Smith, Michael F. Costello, Freya MacMillan, Lisa Moran, Carolyn Ee

**Affiliations:** 1 Monash Centre for Health Research and Implementation, Monash University, Clayton, Vic Australia; 2 NICM Health Research Institute, Western Sydney University, Locked Bag 1797, Penrith, NSW 2751 Australia; 3 School of Women’s and Children’s Health UNSW, Royal Hospital for Women, Barker St, Randwick, NSW 2013 Australia; 4 School of Science and Health and the Translational Health Research Institute, Western Sydney University, Locked Bag 1797, Penrith, NSW 2751 Australia

**Keywords:** PCOS, Barriers, Lifestyle, Weight management, Qualitative

## Abstract

**Background:**

Lifestyle modification targeting weight management is the first-line treatment for women with Polycystic Ovary Syndrome (PCOS) regardless of presenting symptoms. Women with PCOS are more likely to gain more weight compared with women without PCOS, which may be related to barriers in engaging in lifestyle modification. The aim of this study is to explore the experience of women with PCOS in weight management and to determine the facilitators and barriers to lifestyle modifications in women with PCOS.

**Methods:**

Ten women with PCOS participated in focus groups and semi-structured telephone interviews on lifestyle and weight management in PCOS. Discussions were audio-recorded and transcribed verbatim. Thematic analysis of the transcripts was conducted. Thematic analysis of the transcripts were conducted using the method of constant comparison.

**Results:**

Women in the current study attempted a wide range of weight loss interventions, but had difficulties losing weight and preventing weight regain. Women felt that having PCOS affected their ability to lose weight and to keep it off. Facilitators to lifestyle modification for weight management were reported as structured approaches such as having balanced meals and support by health professionals, peers, friends or family. Barriers to lifestyle changes in women with PCOS included logistical barriers such as time and cost, motivational barriers including tiredness or feeling unrewarded, environmental barriers such as not having access to safe places to exercise, emotional barriers such as having depressive and defeating thoughts, and relational barriers such as having unsupportive partner or prioritising children’s meal preferences.

**Conclusions:**

Women with PCOS face a number of personal, environmental and social facilitators and barriers to lifestyle modification for weight loss. While many of these are also experienced by women without PCOS, women with PCOS face additional barriers in having low sense of self-confidence and high prevalence of negative thoughts which may impair their ability to maintain efforts in lifestyle modification over the long term. Future research should further explore the impact of the emotional and mental burden of PCOS on the management of weight and other aspects of PCOS. Future lifestyle intervention should also address the psychosocial aspect of PCOS.

## Background

Polycystic Ovary Syndrome (PCOS) is a common endocrine disorder affecting women of reproductive age. About 8–13% of women are diagnosed with PCOS according to the Rotterdam criteria which included hyperandrogenism, oligo- or anovulation, or polycystic ovary morphology [1, 2]. Apart from the reproductive features included in the diagnostic criteria, women with PCOS also have a greater prevalence of metabolic disorders such as type 2 diabetes, glucose intolerance and metabolic syndrome [3, 4] and psychological co-morbidities including anxiety, depression, body dissatisfaction and lower quality of life [5–9].

Women with PCOS are more likely to be overweight and obese compared with age-matched controls [10]. Excess weight exacerbates all reproductive and metabolic symptoms of PCOS including clinical and biochemical hyperandrogenism, insulin resistance, glucose intolerance and an atherogenic lipid profile [11]. Insulin resistance is a key etiological component in PCOS. Insulin resistance and the resulting hyperinsulinemia produces hyperandrogenemia through its effects on the pituitary, liver and ovaries [12]. Women with PCOS are also at greater risk of weight gain, with a longitudinal study (*n* = 9145) showing women with PCOS gaining an excess of 2.6 kg over 10 years compared with women without PCOS [13]. This is crucial as obesity and weight gain in adulthood is an independent risk factor for chronic diseases including cardiovascular disease, type 2 diabetes and certain types of cancer [14].

Lifestyle modification has been shown to be effective in improving body weight as well as the metabolic, reproductive and psychological features of PCOS [15, 16]. Thus, the International Evidence-Based Guidelines for the Assessment and Management of PCOS suggest lifestyle modification targeting weight management as the first-line treatment for women with PCOS regardless of presenting symptoms [17]. There are no specific diet or exercise recommendations for PCOS [18], therefore the general lifestyle advice from guidelines for the general population of a balanced diet and accumulating 150 to 300 min of moderate intensity exercise weekly applies [17]. In clinical practice, dietary and physical activity goals are tailored to the individual, taking into account weight loss or weight gain prevention goals, medical history, weight history and patient preferences.

Although women with PCOS are more likely to gain more weight compared with women without PCOS, intervention studies have shown that women with or without PCOS are able to lose the same amount of weight when provided with the same support [13, 19]. Women with and without PCOS also had similar resting metabolic rate [20], suggesting that PCOS does not confer a metabolic disadvantage to weight loss. On the other hand, surveys of usual dietary intakes found that women with PCOS consume an additional 250 KJ per day compared with women without PCOS [21]. Women with PCOS are also more sedentary and less active, with an additional half-hour of sedentary time per day and less likely to be physically active (48% vs 64% being physically active) compared with those without PCOS [21, 22]. These small differences in dietary intake and physical activity between women with and without PCOS could account for the greater weight gain in women with PCOS [13]. The reasons for the slightly lower engagement of these lifestyle behaviours in women with PCOS is unclear. On the other hand, it is observed that even within a well-supported program such as lifestyle intervention in RCTs, women with PCOS had a high risk of dropping out, suggesting significant barriers in engaging in lifestyle modification [15]. Considering the key role of lifestyle intervention in the management of PCOS and the difficulty in engaging women with PCOS in lifestyle modification, a better understanding of the perceived facilitators and barriers to lifestyle modification for weight management in women with PCOS is urgently needed.

There have been few qualitative studies conducted in women with PCOS on lifestyle management. As a result, little is known about their experiences in weight management. Past qualitative studies in women with PCOS have focused on experiences on the diagnosis and overall management of PCOS, which yielded valuable insights on the unmet needs of this group in these aspects of their care [23, 24]. However, the experiences of women with PCOS in weight management and lifestyle modification have not been studied. Therefore the aim of this study is to explore the experience of women with PCOS in weight management and to determine the facilitators and barriers to lifestyle modifications in women with PCOS. This investigation is part of a larger qualitative study that assessed the feasibility and acceptability of a method for an RCT on acupuncture and lifestyle for weight management in women with PCOS [25].

## Methods

### Study design

Our study methods have been previously described [25]. We conducted focus groups and semi-structured telephone interviews (offered to women through phone who were unable to attend the focus groups). This study was approved by the Western Sydney Human Research Ethics Committee (H11935/28 Nov 2016). All participants provided written informed consent. If participants provided a telephone interview, the signed consent form was emailed to researchers.

### Participants

Recruitment commenced in December 2016 and was completed in March 2017. Women with PCOS were recruited through social media (paid and unpaid posts on relevant Facebook pages). Inclusion criteria were: self-reported diagnosis of PCOS according to the 2003 Rotterdam Criteria [16]; (BMI) > 25 kg/m^2^; no other endocrine disorders; not pregnant or no pregnancies in preceding 6 weeks and fluent in English, age between 18 and 45 years.

### Data collection and analysis

The focus groups and semi-structured interview questions were developed by CE. Focus groups were moderated by an experienced research officer (BB) while two researchers (CE and LI) observed and took field notes. Semi-structured interviews were conducted by a Health Promotion undergraduate student (LI) experienced in conducting semi-structured interviews. Telephone interviews and focus group discussions were audio-recorded and transcribed verbatim by an independent transcribing service.

Basic demographic data including age, BMI, ethnicity and educational level were collected in a survey. To explore the barriers and facilitators to engaging with these lifestyle management, the interviews and focus groups included questions on past weight loss attempts, knowledge and attitudes towards various different weight interventions, perceived effectiveness and perceived advantages and disadvantages.

Transcripts were circulated to participants for member checking; no clarifications or changes were requested. Thematic analysis of the transcripts was conducted using the method of constant comparison [26]. Three of the investigators (BB, CE and FM) independently coded a subset (20%) of the transcripts. Consensus on codes and subcodes were reached between the investigators through two rounds of discussions. The resulting coding scheme was applied to the rest of the data. The emergent themes were further combined into categories through mind mapping by another investigator (SL). Consensus about the categories was reached through discussion between two investigators (SL and CE). We used Microsoft Excel 2016 Version 15.40 to manage the data.

## Results

Of the 194 enquiries received, 30 women were eligible, and ten participated in the focus groups or semi-structured interviews. Reasons for not being eligible included BMI < 25 (*n* = 29), current use of OCP (*n* = 37), use of insulin sensitisers (*n* = 34), use of gonadotrophins (*n* = 4), unable to attend (*n* = 10), has diabetes (*n* = 7), did not meet PCOS diagnostic criteria (*n* = 6), has another hormonal condition (*n* = 11), pregnant (*n* = 5) within age range (n = 3). Nine of the women who were eligible were uncontactable, eight women were unable to attend focus groups or provide interviews due to distance and/or time constraints and three failed to attend the focus groups and were subsequently uncontactable.

Two focus groups were held, attended by two women and five women respectively. A further five women were unable to attend focus groups and contributed telephone interview data. Focus groups lasted 104 and 117 min respectively while the mean duration of telephone interviews was 21 min (range: 17 to 27 min). By the end of data collection, data saturation in terms of reaching the same main themes had been reached.

Nine out of ten women contributed to the demographic data, while one did not return the survey despite repeated reminders to do so. The majority of women were married, worked full-time, were of European ancestry, and had at least a Bachelor degree qualification. Most women were childless; one woman had four children (Table 1).

**Table 1:** Baseline characteristics of participants

	Mean (SD) or number of participants (%)
Age in years, all participants	36.1 (7.24)
Marital status	
Married	5 (50%)
De facto	2 (20%)
Single	2 (20%)
Missing	1 (10%)^a^
Employment	
Employed full time	9 (90%)
Missing	1 (10%)^a^
Ethnicity/race	
European	5 (50%)
Asian	3 (30%)
Oceanic	1 (10%)
Missing	1 (10%)^a^
Highest educational achievement	
High school - Year 10	1 (10%)
Vocational college	2 (20%)
Bachelor Degree	5 (50%)
Postgraduate	1 (10%)
Missing	1 (10%)^a^
Gross weekly income (annual income)	
$600–799 ($31,200 - 41,599)	1 (10%)
$1000–1249 ($52,000 - 64,999)	3 (30%)
$1500–1999 ($78,000 - 103,999)	5 (50%)
Missing	1 (10%)^a^
BMI, all participants	36.38 (SD 7.8)

*BMI* Body Mass Index

^a^Nine out of ten women contributed to the demographic data, while one did not return the survey despite repeated reminders to do so

### Themes

#### The weight loss journey

Women described a long struggle with their weight, with difficulty in weight management being a key theme. Many spoke of fluctuating weight, typically starting with weight loss from a new intervention followed by a return to baseline, usually at the cessation of the intervention. The weight loss journey was described as a “*rollercoaster ride*”, “*a lifetime of yoyo dieting*”, “*always doing the up and down stuff*”, “*just like a yoyo up and down*”.

*“Did Thai boxing for a while and I'd lost some weight, but then since I moved to Australia somehow I gained 15 kilos again.”*

*“They [meal replacement shakes] were [effective] for while you're having them, but as soon as you stop having them, you just put all the weight back on anyway.”*

Some women gained more weight than what was lost initially, such as Jessica: “*Then you’d beef it back on about three times as much*”.

#### The impact of PCOS on weight

Women felt that having PCOS affected their ability to lose weight and to keep it off. They expressed a self-defeating attitude with the belief that PCOS is a barrier to losing weight. One participant felt that because of PCOS, it took her longer to see results. Eventually, she became discouraged with the lack of results for her efforts and stopped trying. Others similarly became discouraged with the lack of rewards and abandoned their efforts: “*Why aren’t I seeing results, jumping on the scales every day and I do my own head in*”. Some women also expressed frustration about other people seeming to lose weight more easily: “*My husband...he’ll just drop weight like nothing else, and I’ll just be lagging behind*”.

*“Honestly I think at the back of my mind somewhere I don't know that -because I have this condition it's going to take longer for me to reach that goal”.*

*“There were 30 girls in this group and I felt like I was the only one that felt that I had something - up against - I was up against a wall at the first before I even started the fitness, and these girls were just dropping weight.”*

##### Past weight loss experience: “I tried everything”

All participants spoke of their ongoing struggle to lose weight, which had been a challenge for most women since they were teenagers or young adults. Women in the current study attempted a wide range of weight loss interventions. They reported having “*tried everything*”, including diet and exercise, complementary therapies (different types of yoga, Chinese medicine) and medication. A wide range of diet and exercise strategies were reported, including various dietary patterns (CSIRO, eating five meals a day, low glycemic index diets, *“just sort of watching what you eat”,*“*the other one where you don’t eat carbohydrates past two o’clock in the afternoon”*, prepackaged meals (“Lite n Easy”), meal replacements, aqua aerobics, Thai boxing, boot camps and commercial weight loss programs such as Jenny Craig and Michelle Bridges. Some strategies involved receiving support and accountability such as joining the gym, seeing a dietitian or nutritionist or having a personal trainer. One woman thought she had “*explored every diet known to man*” and another said that she thought she “*had tried a lot*” of different exercise regimes. There were a number of interventions that some participants had not tried such as “*those weird diet things, vegan thing”*, and surgery.

*“I think I've looked at everything. I don't think there has been anything in the length of time that I haven't researched at some point and gone, hey this might be a solution.”*

### Facilitators to weight management

Despite these challenges, these women were able to identify what seemed to work for them and what they might need for sustainable weight loss. Several women agreed that accountability was important. “*That daily check-in [with her sister who was also trying to lose weight], the weekly check-in with how much weight we’ve lost. I think that’s what’s helping*”. One participant agreed that the “*only that that worked*” was “*seeing a dietitian and just checking in with her every fortnight*”. Similarly, another agreed that what helped was “*having someone motivating you and keeping you on track and keeping you accountable*”.

*“But just going to the gym by yourself is really hard to get yourself motivated and keep on track because you don't have anyone pushing you except for yourself, but if you're not seeing results, you let yourself get down”.*

There was a consensus that diet and exercise were effective interventions. Women appeared to have some knowledge about effective diet and exercise regimes:

*“I definitely feel that the balanced meal is the one that is most effective to me.”*

*“I think out of all of them the biggest weight loss I had was definitely…your breakfast was your bigger meal. Then your lunch was smaller. Then dinner was the shake or just like a normal protein shake with milk. You had steamed vegetables or you had salad. That was probably the most effective. …for a good three to six months. So, I [was] able to do that and that was okay.”*

*“It [personal training] was very effective when I was using it, yeah.”*

### Barriers to weight management

Participants were quick to articulate the many barriers that prevented sustainable weight loss mostly through lifestyle modification, and there were many more perceived barriers than there were facilitators. A variety of general personal or motivational factors were seen to be major barriers, such as tiredness, disliking the food choices and the “mundaneness” of a restricted diet. For example, Jessica enjoyed cooking, referring to it as a “*de-stressor*”, and therefore disliked having restrictions on what she could prepare and eat. Feeling discouraged by the lack of results identified earlier as their experiences with weight management were also cited as a barrier to weight loss.

There were also general environmental or systemic barriers such as not having a suitable place to exercise, and this intersected with the feeling of embarrassment about being seen outdoors or in a gym. The latter was related to negative thoughts such as about feeling depressed and unable to motivate self.

*“That’s certainly the case especially sometimes when you go to the gym the whole atmosphere in the gym is like - I don't know, you see all those skinny models wearing crop-tops and stuff and all that. I don't know, sometimes I just feel, I don't know, negative and then sometimes I want to go and lift weights, but I don't want to go in that section. I dread going to that section. I've never been to that section because - I want to but I won't.”*

*“Often exercise I don't want to do it outside of the house at all. I don't want people to see me even walking down the street. Often, I just want to stay at home. So, I guess it's either embarrassment or depression from being overweight that just - the thought of actually - once I've got myself in the gear I'm fine, but that effort to get into it and really push myself I don't have that”*

There were also logistical issues such as the prohibitive cost of personal training, meal delivery services or seeing a dietitian regularly.

*“I think for me personally at times there have been some financial constraints as well, to be honest. Going and seeing the personal trainer [I'll go and see] him every week for $80 an hour or I might go to a dietitian or a nutritionist as well on a regular basis, but it does sometimes cost you a whole lot of money.”*

Work commitments were a barrier, for example, one woman found that a diet that required her to eat five times a day worked well for her but was not sustainable once she started fulltime work.

Sometimes several factors would conspire to prevent exercise, such as time, environmental factors, and being tired after work:

*“I don't live in the greatest suburb in the world and I certainly don't like walking there late at night and I don't [inaudible] after work purely because I have a very long day and my job's quite brain-draining*.*”*

One woman, who had children, felt that specific diets are challenging to maintain in a family environment. She knew that meal replacements and Weight watchers worked for her but said:

*“So, it's really hard to just sit down and go I'm just going to make my food and then still have to accommodate for having kids. So that’s one of the issues. The shake situation was the same because you sat down to a shake. But how do you sit down to have a shake when you've got four and five-year-olds who are looking at you going, well why are you having that and I'm not having food. Trying to explain that situation.”*

Partners were also cited as a barrier. One participant’s husband tended to reward her with food. Another said that “*the house has to change the lifestyle”.* She struggled with having a husband who did not worry about weight loss and said

*“I feel like Monday to Friday when I'm at work I'm pretty good with my food, but on weekends when I'm with him it's just ridiculous.”*

## Discussion

This paper describes the experiences of women with PCOS in managing their weight, and their perceived facilitators and barriers in lifestyle modification for weight loss. We found that women with PCOS were generally experienced in attempting weight loss but had difficulties losing weight and preventing weight regain. Facilitators to lifestyle modification for weight management were reported as structured approaches such as having balanced meals and support by health professionals, peers, friends or family. Barriers to lifestyle changes in women with PCOS included logistical barriers such as time and cost, motivational barriers including tiredness or feeling unrewarded, environmental barriers such as not having access to safe places to exercise, emotional barriers such as having depressive and defeating thoughts, and relational barriers such as having unsupportive partner or prioritising children’s meal preferences. While many of these are also experienced by women without PCOS, some barriers such as tiredness and emotional barriers may be exacerbated by the presence of PCOS.

### Impact of PCOS on weight

Women with PCOS believed PCOS deters weight loss and predisposes them to regain weight following weight loss. As a result, weight cycling was a common experience in women with PCOS in the current study. Difficulty losing weight, weight cycling and unexplained weight gain were also reported by other women with PCOS in previous studies [8, 27–29]. This was further supported by a longitudinal study which also found women with PCOS having greater weight gain than age-matched women without PCOS [13]. The physiological basis supporting greater weight gain in PCOS is unclear. This may be related to the greater energy intake and lower physical activity in women with PCOS [21]. The slightly higher dietary intake may be due to dysfunction in appetite regulation as women with PCOS had impaired levels of appetite hormones such as cholecystokinin and ghrelin [30, 31]. Apart from these behavioural factors with possible upstream causes, women with PCOS do not appear to have a metabolic basis for being more resistant to weight loss [20]. Weight regain following weight loss can have a detrimental effect on the mental well-being of women with PCOS including feelings of guilt, low self-esteem and poor self-image [8, 28, 29]. These can further erode the motivation to maintain efforts to manage weight, preventing recovery from the relapse.

### Past weight loss attempts

We also found that women with PCOS have a broad range of experience in weight management, including diet and exercise, medication and alternative therapies. This is consistent with a previous study which found that women with PCOS engaged in a wide range of weight management practices and were more likely to attempt most of the practices compared with women without PCOS [32]. However, most of these attempts appeared to be self-initiated with very little or no support by health professionals [33, 34]. The involvement of a dietitian is unlikely according to the low referral rate of PCOS patient to dietitians, resulting in women with PCOS seeking alternative sources of advice and support for lifestyle modification [34, 35]. It is known that certain behaviour change techniques are essential for successful lifestyle modification [36]. These techniques are unlikely to be consistently applied without constant monitoring and feedback. This may explain the lack of success and frustration observed in the self-initiated, unsupported weight loss attempts in women with PCOS.

### Facilitators and barriers to lifestyle modifications

The facilitators to lifestyle modifications reported by women with PCOS in the current study are similar to that reported by women without PCOS in previous studies [37, 38]. In the current study, women with PCOS reported that structured approaches such as having a balanced meal or a bigger breakfast were helpful in their weight loss attempts. The previous study in young women without PCOS similarly found that structured dietary advice (e.g. structured meal plan with specified portion sizes) were more effective in weight loss when compared to qualitative advice (e.g. “reduce fat intake”) [37]. In addition to specific dietary strategies, women in the current study identified that accountability and peer support as a facilitator. These were also reported by young women without PCOS in previous studies [38, 39]. However, women without PCOS have also previously reported perceiving the lifestyle changes as enjoyable and fun, indicating intrinsic motivation for their efforts [39]. This was not reported by the women in the current study, in whom lifestyle modifications were only perceived as a means of weight loss, which represents an extrinsic motivation in the form of an external reward. Intrinsic motivation such as the enjoyment of the activity has been associated with long term weight loss maintenance [40], and the lack of this facilitating factor in women with PCOS may explain their lack of long term success with any strategy for weight management.

Women in the current study reported a range of barriers to lifestyle modification. Most of these barriers were also reported by young women without PCOS, with the key barriers being work commitments, time, cost and difficulty with children [22, 29, 41–43]. Personal barriers such as disliking the taste of certain foods were also similar across women with and without PCOS in current and previous studies [41, 44]. Environmental barriers such as not having access to opportunities for physical activity were reported as one of the barriers in women of the current study and in young women without PCOS in other studies [39, 41]. Having an unsupportive partner, as cited by women in the current study, was similarly identified as a barrier by women without PCOS [41, 44, 45]. Considering the similarities in barriers between young women with and without PCOS, these barriers to lifestyle modifications in women with PCOS may be related to their life-stage more so than PCOS itself.

Certain barriers to lifestyle changes were unique to women with PCOS. Women with PCOS in the current study reported tiredness as a barrier to lifestyle medication. Feelings of tiredness and fatigue have been previously described as one of the experiences of living with PCOS [8, 22, 29]. It is unclear if this was relating to the mental-burden of dealing with PCOS symptoms, as women with PCOS have previously reported intense pre-occupation with certain symptoms such as hirsutism or menstrual irregularity that led to an inability to plan and problem-solve [8]. Women in the current study also felt embarrassed to exercise at the gym or outdoors. Although overweight or obese women without PCOS have also cited feeling embarrassed as a barrier to exercise [45], evidence suggests that women with PCOS have greater body dissatisfaction than women without PCOS [6]. These feelings of inadequacy were confirmed by previous studies in women with PCOS which reported feelings of low self-confidence, self-worth and self-esteem; feelings of being inferior and abnormal compared with peers and feelings of hopelessness [6, 8, 9, 28, 29]. Adding to these negative self-assessments are the prevalent depressive or negative thoughts, which were cited as a barrier to maintain lifestyle efforts in the current study. This is consistent with previous studies reporting higher depression scores in women with PCOS [6, 9]. This overall negative outlook is also consistent with the lower perceived quality of life experienced by women with PCOS [5, 7, 8]. As self-efficacy is an important predictor of lifestyle behaviour particularly in women with higher BMI [46, 47], the low sense of agency over health in women with PCOS could be detrimental to the long term maintenance of lifestyle behaviours.

The main strength of this study is the description of facilitators and barriers to lifestyle modification for weight management in women with PCOS. To our knowledge, this is the first study exploring this in an in-depth manner, with the only other qualitative study in lifestyle barriers and facilitators exploring the experience of women with PCOS on a commercial very-low-calorie diet [29]. This study was conducted and reported according to the Consolidated criteria for reporting qualitative studies (COREQ) guidelines [48] which ensures sound and logical conduct of the research process that contributes to dependability. Data source triangulation and independent coding by several reviewers enhances credibility of the findings. Participants characteristics were provided to assist readers to decide on transferability of the findings to other populations and direct quotes are provided to illustrate the themes. Interviewers’ and focus group facilitator’s characteristic were described to address reflexivity.

The current study has several limitations. First, the sample size is small, including one focus group which contained two participants. However thematic saturation was achieved in the overall sample and the themes were agreed upon by two reviewers, providing support for internal consistency. Additionally, the use of data source triangulation (focus group and interview data) allowed us to gain broader perspectives on the topic and enhances the credibility of the study.

Second, the interviews were conducted in English without the presence of interpreters, which limited our ability to capture the experiences of those who do not speak English. The findings of this paper should be considered with these limitations in mind.

## Conclusion

Women with PCOS attempt a wide range of weight management practices but found achieving and maintaining weight loss challenging. This may be related to the lack of professional support in their attempts. Women with PCOS face a number of personal, environmental and social facilitators and barriers to lifestyle modification for weight loss, which may be related to their life stage. Women with PCOS face additional barriers in having a low sense of self-confidence and high prevalence of negative thoughts which may impair their ability to maintain efforts in lifestyle modification over the long term. Future research should further explore the impact of the emotional and mental burden of PCOS on the management of weight and other aspects of PCOS. Further research is needed to develop a lifestyle intervention that also addresses the psychosocial aspect of PCOS.

## Data Availability

The datasets used and/or analysed during the current study are available from the corresponding author on reasonable request.

## Abbreviations

BMI: Body mass index
PCOS: Polycystic ovary syndrome
